# Germline heterozygous exons 8–11 pathogenic *BARD1* gene deletion reported for the first time in a family with suspicion of a hereditary colorectal cancer syndrome: more than an incidental finding?

**DOI:** 10.1186/s13053-023-00246-4

**Published:** 2023-01-28

**Authors:** Sergio Carrera, Ana Belén Rodríguez-Martínez, Intza Garin, Esther Sarasola, Cristina Martínez, Hiart Maortua, Almudena Callejo, Abigail Ruiz de Lobera, Alberto Muñoz, Nagore Miñambres, Pablo Jiménez-Labaig

**Affiliations:** 1 Hereditary Cancer Genetic Counseling Unit- Medical Oncology Department, Cruces University Hospital, Plaza de Cruces S/N. 48903, Baracaldo, Bizkaia Spain; 2 Molecular Genetics Laboratory, Basurto University Hospital, Bilbao, Spain; 3 Molecular Genetics Laboratory, Cruces University Hospital, Baracaldo, Spain; 4 Medical Oncology Department, Cruces University Hospital, Baracaldo, Spain

**Keywords:** Colorectal cancer, Hereditary, *BARD1*, Pathogenic variant, Deletion, Amsterdam clinical criteria, Familial colorectal cancer type X syndrome, Cancer genetic counseling

## Abstract

**Background:**

Colorectal cancer (CRC) is a highly prevalent disease in developed countries. Inherited Mendelian causes account for approximately 5% of CRC cases, with Lynch syndrome and familial adenomatous polyposis being the most prevalent forms. Scientific efforts are focused on the discovery of new candidate genes associated with CRC and new associations of phenotypes with well-established cancer-related genes. BRCA1-associated ring domain (*BARD1*) gene deleterious germline variants are associated with a moderate increase in the relative risk of breast cancer, but their association with other neoplasms, such as CRC, remains unclear.

**Case presentation:**

We present the case of a 49-year-old male diagnosed with rectal adenocarcinoma whose maternal family fulfilled Amsterdam clinical criteria for Lynch syndrome. Genetic test confirmed the presence in heterozygosis of a germline pathogenic deletion of exons 8–11 in *BARD1* gene. The predictive genetic study of the family revealed the presence of this pathogenic variant in his deceased cancer affected relatives, confirming co-segregation of the deletion with the disease.

**Conclusions:**

To the best of our knowledge, this is the first published work in which this *BARD1* deletion is detected in a family with familial colorectal cancer type X (FCCTX) syndrome, in which the clinical criteria for Lynch syndrome without alteration of the DNA mismatch repair (MMR) system are fulfilled. Whether this incidental germline finding is the cause of familial colorectal aggregation remains to be elucidated in scientific forums. Patients should be carefully assessed in specific cancer genetic counseling units to account for hypothetical casual findings in other genes, in principle unrelated to the initial clinical suspicion, but with potential impact on their health.

## Background

CRC is a highly prevalent disease in developed countries. In the 27 countries of the European Union (EU), CRC was estimated to account for 12.7% of all new cancer diagnoses and 12.4% of all cancer deaths in 2020, being the second most common cancer after breast cancer and the second most common cause of cancer mortality after lung cancer [1, 2].

Like other complex diseases, CRC is caused by external factors and genetic susceptibility factors. Different studies point to up to 35% of inherited genetic factors involved, of which an estimated 5% correspond to a Mendelian inheritance pattern [3]. Within the latter inheritance model, Lynch syndrome stands out as an example of HNPCC (hereditary non-polyposis colorectal cancer) [4]. Among families in which Amsterdam I clinical criteria for Lynch syndrome are fulfilled, but neither deficient-MMR (dMMR) proteins nor high microsatellite instability (H-MSI) are found, FCCTX is established as a definition of a syndrome in which inherited genetic factors are suspected to be involved, but are not yet clearly defined [5, 6]. These families have three or more relatives with CRC, two or more consecutive affected generations and at least one case of CRC diagnosed before the age of 50. Efforts to find heritable genetic causes in the context of proficient-MMR (pMMR) non-polyposis CRC remain enormous to date, but without obvious evident clear conclusions. In this context, genes involved in DNA repair of double-strand breaks (DSBs) are among the candidates to be studied [7].

*BARD1* gene is located on chromosome 2 (2q34-35). It is composed of 11 exons and encodes a protein of 777 amino acids. It is divided into the following domains: a RING-finger domain in the N-terminal region, which interacts directly with the BRCA1 protein, two conserved tandem domains in the C-terminal region of BRCA1 (BRCT) and four tandem ankyrin repeats. BRCA1-BARD1 forms a tumor suppressor complex that interacts with DNA to repair DSBs through homologous recombination (HR) [8]. In addition, the *BARD1* gene has many BRCA1-independent functions, such as induction of apoptosis through p53 stabilization. The latter function is mainly mediated by its BRCT1 and BRCT2 domains, which correspond to exons 8–11 [9, 10]. BRCT domains are also critical in poly ADP-ribose (PAR) signaling in response to DNA damage, through their binding to the ADP-ribose within PAR, mediating BRCA1 recruitment [11]. Furthermore, *BARD1* has been shown to behave as an oncogene, as reported in studies in which overexpression of spliced isoforms of BARD1 have been detected in tumor tissues [12].

The *BARD1* gene has been recently established as a moderate risk gene for hereditary breast cancer, particularly in triple-negative breast cancer [13, 14]. In contrast, its association with increased risk for other cancers, such as CRC, remains a matter of debate. Although the number of families with CRC aggregation in which deleterious variants in the *BARD1* gene continues to increase, data are scarce and no unequivocal association has been found [15, 16].

## Case presentation

We present the case of a 49-year-old Caucasian man who debuted with rectal bleeding as the first symptom. Colonoscopy was performed and revealed the presence of a tumor in the middle rectum. The biopsy was compatible with an intestinal adenocarcinoma and immunohistochemistry (IHC) for MMR proteins was proficient (pMMR). A contrast-enhanced whole body computed tomography (CT) scan was carried out, which revealed no signs of any distant lesions. A pelvic magnetic resonance imaging (MRI) scan was also performed, which confirmed the presence of a locally advanced rectal tumor (TNM clinical classification cT3N2aM0, stage IIIB) [17]. The patient was treated with concomitant neoadjuvant radiotherapy and chemotherapy (capecitabine), obtaining a partial radiological response. Subsequently, the patient underwent low anterior resection (LAR) with total mesorectal excision (TME) and ileostomy, and the pathological specimen reported the presence of a G2ypT3ypN0 (0/12) LVI- (lymphovascular invasion), CRM- (circumferential resection margin), R0 intestinal adenocarcinoma of the rectum (TNM stage IIA pathological classification) [17]. Subsequently, the patient received six cycles of adjuvant chemotherapy with capecitabine, with good tolerance and no delays between dose administrations.

The patient was referred to our Hereditary Cancer Genetic Counseling Unit for further studies due to the age of presentation of the cancer and a significant family history. The complete physical examination revealed no typical phenotypic features suggestive of any specific syndrome. Polymerase chain reaction (PCR) analysis of microsatellite instability (BAT-25, BAT-26, NR-21, NR-24 and NR-27) performed on the tumor sample was stable (MSS). The patient´s family history was particularly striking: he had a 60-year-old sister who was diagnosed with pMMR endometrial adenocarcinoma at the age of 56. His 86 year old father was diagnosed at the age of 85 with colon adenocarcinoma with loss of MLH1-PMS2 in the IHC, *BRAF* V600E wild-type (WT) and hypermethylation of the *MLH1* promoter, strongly suggesting a sporadic origin. No other cases of cancer were reported in first and second-degree relatives in the paternal line. His mother, who was diagnosed with colon cancer at the age of 53, died at the age of 55. One of his maternal aunts died of colon cancer at the age of 50, another maternal aunt died of an extra-uterine pregnancy at age 29, another maternal aunt died at age 80 and had triple negative breast cancer at age 74, and his maternal uncle died at age 81 and was diagnosed with colon cancer at age 79. His maternal grandmother died at 60 from a probable cancer of unknown origin and his maternal grandfather died at 80 from a myocardial infarction. Overall, maternal history was suggestive of an autosomal dominant pattern of inheritance.

## Methodology

Given the suspicion of a FCCTX syndrome, in which the clinical criteria for Lynch syndrome were met without alteration of the MMR system and taking into account the maternal family history, compatible with an autosomal dominant inheritance pattern, we offered the study of a panel of cancer susceptibility genes by Next Generation Sequencing (NGS), after the pre-test consultation and obtaining informed consent. During pre-test consultation, special emphasis was placed on explaining the genes related to clinical suspicion, but the possibility of detecting incidental findings in other genes included in the panel was also explained.

### Sample collection and DNA isolation

Peripheral blood samples (PB) were collected following the standard procedure for diagnostic testing after written informed consent, in accordance with the current revision of the Helsinki Declaration. Genomic DNA was extracted (QIASymphony, Qiagen), quantified (Qubit dsDNA High Sensitivity kit and Nanodrop, Thermo Scientific) and quantity and quality checked (LabChip GX Touch 24 analyzer, Perkin Elmer) according to corresponding NGS protocol’s guidelines.

### Libraries set-up and sequencing

Sequencing libraries were prepared using the CE-IVD SOPHiA HCS v2 kit, with automated procedure implemented on Zephyr G3 NGS (Perkin Elmer). Individual libraries were quantified and quality control analysis was done as described before.

### Data analysis and variants interpretation criteria

The sequencing data were simultaneously processed for single nucleotide variants (SNVs), indels, and copy number variations (CNVs) using the last version available of Sophia DDM software. Data analysis and variant interpretation were limited to virtual panels of actionable genes, in accordance with the informed consent expressed and signed by the patient. For the assessment of colorectal risk, the following gene panel was analyzed: *MLH1, MSH2, MSH6, PMS2, EPCAM, POLE, POLD1, MUTYH, APC, AXIN2, BMPR1A, CHEK2, GREM1, MLH3, MSH3, NTHL1, PTEN, SMAD4, STK11* and *TP53*.

Genetic variants annotations were also integrated with data present in literature and specific databases such as Insight-group [18], Leiden Open source Variation Database [19], ClinVar [20], 1000 Genomes Project [21], ExAC [22], dbSNP [23] and The Genome Aggregation Database [24].

Variants were reported using the international standard HGVS nomenclature and classified into five categories: pathogenic (P), likely pathogenic (LP), variant of uncertain significance (VUS), likely benign (LB) and benign (B), according to the American College of Medical Genetics and Genomics (ACMG) criteria [25].

### NGS variants confirmation

Genetic variants interpreted as pathogenic or probably pathogenic were confirmed by Sanger sequencing or multiplex ligation-dependent probe amplification (MLPA) (MRC-Holland), as appropriate. MLPA data were analyzed with the latest version of Coffalyser.Net software (MRC-Holland).

#### CNV extension analysis

In order to confirm and establish detected CNV extension, a comparative genomic hybridization oligonucleotide microarray (aCGH), containing around 60,000 probes distributed throughout the genome (60 K from Agilent qChip®Post CM kit; qGenomics) was used. Test sample was hybridized against a sex-matched reference (human reference DNA, Agilent Technologies) and obtained data were analyzed using Cytogenomics 4.0.3.12 and qGenviewer software (analysis parameters: algorithm ADM2 ≥ 6.0; abs (log2ratio) ≥ 0.25; probes ≥ 3).

## Results

**Fig. 1:**
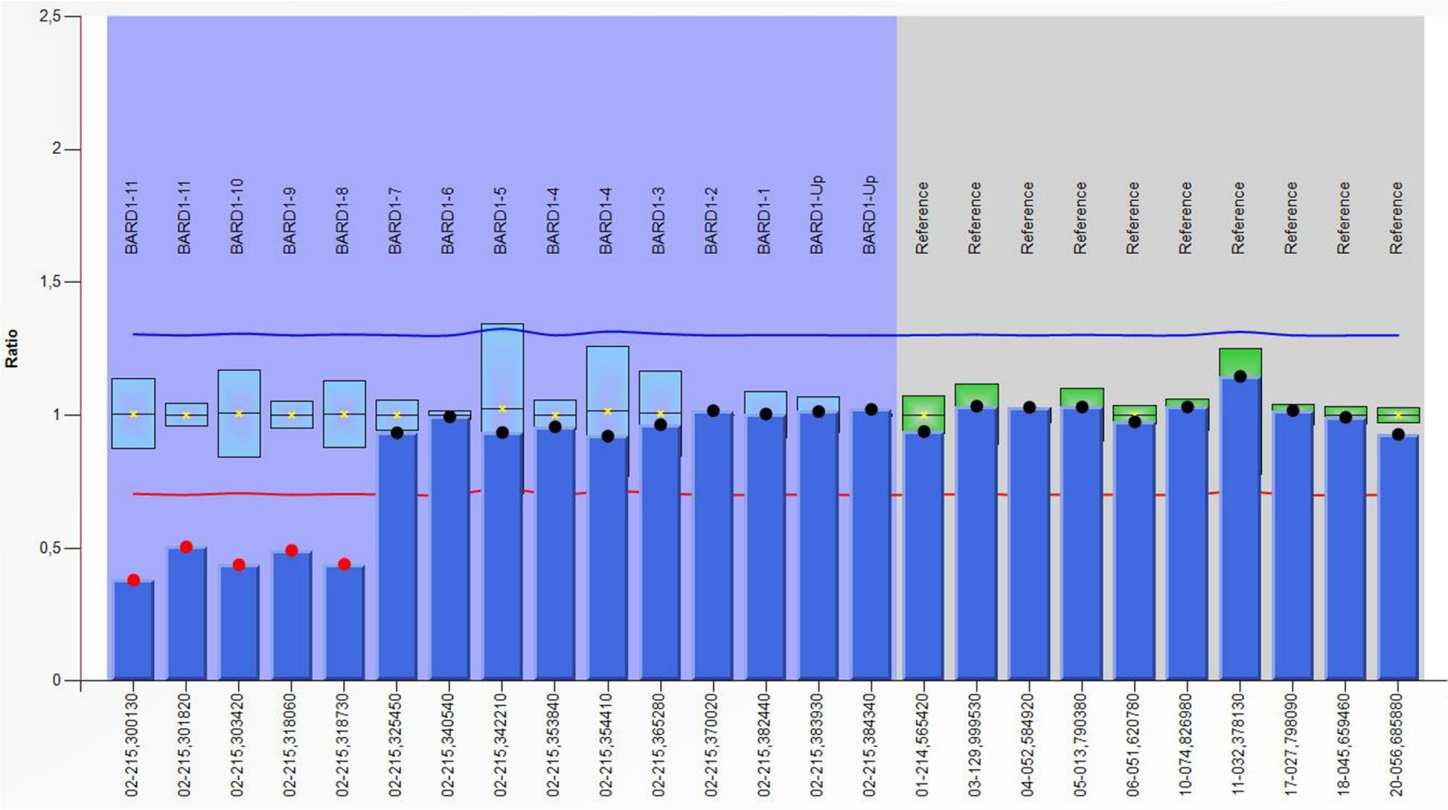
Multiplex Ligation-Dependent Probe Amplification (MLPA) analysis: One hundred nanograms of genomic DNA of the proband and his relatives were analyzed for CNV confirmation/detection in *BARD1* gene with SALSA MLPA P489 probemix (MRC Holland, Amsterdam, The Netherlands) according to manufacturer’s instructions on an ABI3500 Genetic Analyzer (Thermo Fisher Scientific). Data were analyzed with Coffalyser.NET software (MRC Holland)

No deleterious variants were detected in well-established colorectal cancer susceptibility genes. Unexpectedly, germline genetic analysis of the rest of 84 genes included in the SOPHIA Hereditary Cancer Solution revealed the presence of a heterozygous deletion of exons 8–11 in *BARD1* gene (NM_000465.2). The deletion was confirmed by MLPA (Fig. 1). The aCGH analysis confirmed the absence of a larger deletion affecting *BARD1* that could involve surrounding morbid genes. To the best of our knowledge, this CNV—GRCh36 chr2: g.(¿_214727148)_(214745750_?)del—has not been reported in specific databases or population studies. This large deletion is predicted to cause an out-of-frame RNA that results in incorrect translation of the protein, generating a premature stop codon and involving the loss of critical functional domains. By virtue of its nature and following ACMG, this CNV is considered as likely pathogenic/pathogenic [25, 26].

**Fig. 2:**
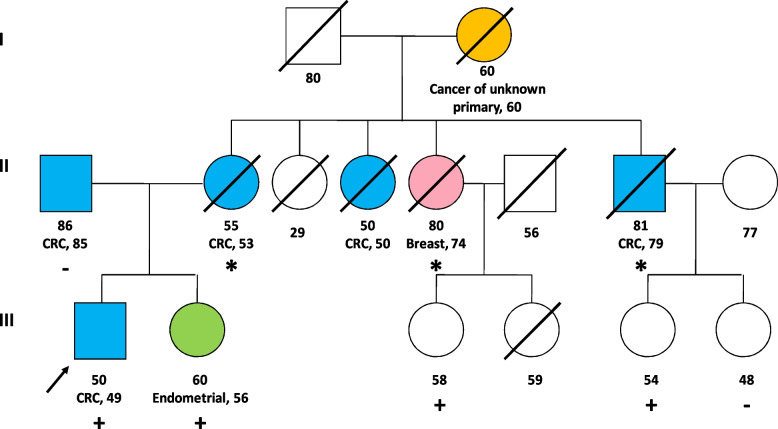
Family pedigree. The arrow denotes the proband. Blue color: colorectal cancer (CRC) affected. Green color: endometrial cancer affected. Orange color: cancer of unknown primary affected. Pink color: breast cancer affected. Plus ( +) and minus (-) sign represent carrier status of tested family members: *BARD1* pathogenic deletion carrier is represented by plus ( +) sign, and no pathogenic deletion carrier is represented by minus (-). Obligate carrier status is represented by an asterisk (*)

Subsequently, we performed predictive genetic tests to his relatives. His sister, diagnosed with endometrial pMMR adenocarcinoma at the age of 56, was also carrier of the variant. His father was found to be not carrier of the variant and his mother died of colon cancer at the age of 55 so, in order to assess maternal inheritance and discard the possibility of gonadal mosaicism, we offered predictive test to the offspring of his maternal uncles and aunts, all deceased, if applicable. One daughter of his maternal uncle who died because of colon cancer at the age of 81 and another daughter of his deceased maternal aunt who suffered from triple negative breast cancer at the age of 74 were also carriers of the variant. These results confirmed that his mother and the mentioned maternal uncle and aunt, all cancer affected, were obligate carriers of the variant. These results are consistent with clinical suspicion, as on the one hand, the variant appears to cosegregate with the phenotype and, on the other hand, pathogenic variants in the *BARD1* gene have been associated with an increased relative risk of triple negative breast cancer [13, 14]. Figure 2 shows the family pedigree.

## Discussion and conclusions

A comprehensive review of the medical literature and available databases was performed, confirming that germline deletion of *BARD1* exons 8–11 has not been published to date. Deleterious variants affecting the BRCT domains of the *BARD1* gene have been previously reported in patients with breast or ovarian cancer [27]. Deletions of *BARD1* exons 7–8 and 7–11 were previously described in two patients diagnosed with ovarian and triple negative breast cancer, respectively, in a Spanish study [28]. Another deletion in the *BARD1* gene affecting its BRCT domains was also reported in a patient diagnosed with ovarian cancer [29]. A more recently published study in families with breast-ovarian cancer aggregation revealed the presence of five different large deletions in the *BARD1* gene in five breast cancer patients. In two of the cases, there was a confirmed history of CRC among their relatives, but no data on cosegregation were provided [30].

Published cases of CRC patients with deleterious variants in the *BARD1* gene are scarce [15]. The pathogenic variant c.1811-2A > G of *BARD1*, which is predicted to cause exon 9 skipping, has been published in a family with CRC aggregation [16]. Another pathogenic *BARD1* variant, also affecting BRCT domains, has been described in a woman diagnosed with CRC [31]. In another study, the authors reported three patients diagnosed with CRC before the age of 50 years with variants of unknown significance (VUS) in the *BARD1* gene, one of them in BRCT domains [32]. A deletion affecting exons 5–6 of the *BARD1* gene has also been detected in a patient diagnosed with rectal adenocarcinoma in a study involving 1,058 unselected CRC cases [33].

Several studies have attempted to detect potential new candidate genes associated with inherited genetic susceptibility to CRC [34]. *BARD1* is among the genes with a hypothesized but unconfirmed association with CRC [7, 27, 31]. Functional and larger population-based studies are needed to confirm the possible association of *BARD1* with an increased relative risk of CRC [35]. In this line of research, some studies have attempted to demonstrate that either the expression of the full-length BARD1 protein and/or N-terminal and C-terminal BARD1 epitopes in colon tumor tissues is associated with better prognosis [12, 36, 37], suggesting that BARD1 regulation is an important pathway in colon cancer.

To the best of our knowledge, there are no published references of the deletion of exons 8–11 in the *BARD1* gene in a patient diagnosed with CRC in the context of a family with FCCTX syndrome. Although we cannot demonstrate unequivocal causality between this genetic finding and the cancer aggregation described in our family, predictive studies performed in relatives confirmed maternal inheritance of the deletion, its cosegregation with the phenotype and its presence in the deceased maternal aunt diagnosed with triple negative breast cancer, which is consistent with current scientific evidence [13, 14].

In this case report we describe for the first time the presence of a deletion of exons 8–11 in the *BARD1* gene in a young male diagnosed with adenocarcinoma of the rectum in the context of a family with CRC aggregation and a confirmed case of triple negative breast cancer in a relative. For the moment, the sole factor of presence or absence of the deletion will not allow us to estimate the risk of CRC in family members. However, knowledge of it may allow us to do so in the future, if a cause-effect relationship is eventually established. Regardless of whether this germline deletion of *BARD1* is a chance or causal finding, or neither, detection of this variant will allow us to provide appropriate genetic counseling on familial breast cancer risk. In addition, this case reflects the importance of appropriate genetic counseling for patients and their relatives, who should be previously informed of the possibility of detecting relevant findings with potential repercussions on their health, in principle, unrelated to the clinical suspicion that motivated the germline study.

## Data Availability

Not applicable.

## Abbreviations

CRC: Colorectal cancer
*BARD1*: BRCA1-associated ring domain
FCCTX: Familial colorectal cancer type X
HNPCC: Hereditary nonpolyposis colorectal cancer
MMR: Mismatch repair
dMMR: Deficient mismatch repair
pMMR: Proficient mismatch repair
H-MSI: High microsatellite instability
MSS: Microsatellite stable
IHC: Immunohistochemistry
CT: Computed tomography
NGS: Next Generation Sequencing
MLPA: Multiple Ligation-dependent Probe Amplification
CNVs: Copy number variants
ACMG: American College of Medical Genetics and Genomics
MRI: Magnetic resonance imaging
LAR: Low anterior resection
TME: Total mesorectal excision
LVI: Lymphovascular invasion
CRM: Circumferential resection margin
WT: Wild type
PCR: Polymerase chain reaction
PB: Peripheral blood
HGVS: Human Genome Variation Society
HR: Homologous recombination
PAR: Poly ADP-ribose
DSBs: Double-strand breaks
SNVs: Single nucleotide variants
aCGH: Array comparative genome hybridization
